# Case of Amputation of Both Thumbs by Machinery, the Tendon of the Flexor Longus Pollicis Being Drawn off as High up as Its Attachment to the Muscle

**Published:** 1838-12-19

**Authors:** Henry H. Smith

**Affiliations:** Resident Surgeon


*Case of Amputation of both Thumbs by machinery, the tendon of the Flexor Longus Pollicis being drawn off as high up as its attachment to the muscle.*

William H—, aged 22 years, while engaged in casting a band off a drum in a factory, had his thumbs entangled in it, and both taken off. The divided vessels were secured, and the wound dressed by a surgeon in the neighborhood, and he was afterwards sent to this hospital.

He was admitted October 17th, with a lacerated wound at the metacarpal joint of each thumb—the bones were broken above the joint, and the rest of the hand was uninjured. He brought with him the thumb of the left hand, to which the tendon of the flexor longus pollicis was attached; the tendon was of full length, and had shreds of the muscle adherent to it. The wounds were drawn together with adhesive strips; dry lint applied over this, and the hands loosely bandaged on splints extending from beyond the fingers to the elbow. The patient was placed in bed; the limb elevated, and lead water kept constantly applied by means of a syphon: an opiate.

The dressings and applications were continued till October 23d; upon removing them, but little inflammation was found, and the wounds had not united: simple dressings.

*25th.*—Wounds suppurating; slight slough of cellular substance on the right thumb; left suppurating; no pain in the arm, and no appearance of a sinus in the course of the tendon. Adhesive straps removed; wounds poulticed, splints continued.

This dressing was continued until October 28th, when the left thumb presented the appearance of a healthy ulcer, and was treated accordingly. The sloughing of the right thumb continued until November 2d, and was dressed with the fermenting poultice. The sloughs separating, a simple poultice was applied, and the patient allowed to walk about.

*Nov. 7th.*—Right thumb granulating and closing up rapidly; the left nearly well; dressed only with cerate, confined by a strip; all the motions of the left hand are perfect, even to the stump; splint continued to the right; general health perfect.

*16th.*—Left thumb healed to the size of a sixpence; a small piece of bone removed from the right, which is also healing rapidly; dressed with mucilage.

*20th.*—Left thumb entirely well; the other has a small ulcer still on it; motions in right hand perfectly good.

*December 6th.*—Patient entirely well; motion in the remaining joint of each thumb; feels nothing wrong in the left arm; motion of all his joints perfect. This patient was not discharged until the 10th, owing to circumstances not connected with his case. The time occupied in the treatment was fifty days, and he at no time had a bad symptom.

